# Severe SARS-CoV-2 Infection: A Multisystem Inflammatory Syndrome in Moroccan Children

**DOI:** 10.7759/cureus.12991

**Published:** 2021-01-29

**Authors:** Amal Haoudar, Nabila Chekhlabi, Madiha Eljazouly, Chafik El Kettani, Nezha Dini

**Affiliations:** 1 Anesthesia and Critical Care, Cheikh Khalifa International University Hospital, Mohammed VI University of Health Sciences, Casablanca, MAR; 2 Department of Pediatrics, Cheikh Khalifa International University Hospital, Mohammed VI University of Health Sciences, Casablanca, MAR; 3 Dermatology Unit, Cheikh Khalifa International University Hospital, Mohammed VI University of Health Sciences, Casablanca, MAR; 4 Department of Pediatrics, Mohammed V University, Faculty of Medicine and Pharmacy of Rabat, Rabat, MAR

**Keywords:** sars-cov-2, kawasaki disease, multisystem inflammatory syndrome, coronary artery dilation

## Abstract

Until October 2020, children seem to have a mild form of severe acute respiratory syndrome coronavirus 2 (SARS-CoV-2) infection in Morocco. Since April 2020, a new entity of multisystem inflammatory syndrome in children associated with SARS-CoV-2 infection was reported in England, Italy, France, and the United States. This syndrome has similarities with the most frequent vasculitis in children, Kawasaki disease. Here, we describe the features and outcome of the first five cases of Kawasaki-like multisystem inflammatory syndrome in Moroccan children triggered by the SARS-CoV-2. The median age of the children was 7.8 years; three of them were boys. Criteria for Kawasaki disease were met in all of them with a complete presentation. Three patients required admission to a critical care unit with multi-organ failure in one of them, but no mortality occurred. They all received intravenous immunoglobulin (IVIG), a high dose of aspirin, methylprednisolone, and supportive therapy. Coronavirus 2019 disease (COVID-19) infection in children may be life-threatening; rigorous monitoring for several weeks is required in any positive child or living in a family cluster.

## Introduction

The World Health Organization declared a global pandemic on March 11, 2020. Fewer cases of coronavirus disease 2019 (COVID-19) have been diagnosed in children than in adults, and most of them have been mild. In Morocco, children got COVID-19 infection from family members, and they seem to have a milder illness than adults, with mild symptoms and a good short-term prognosis [1]. However, since mid-April 2020, multiple case clusters of what has been termed multisystem inflammatory syndrome in children (MIS-C) have been reported from different countries and, as of October 1, 2020, there were 1,027 cases reported in the United States with a total of 20 deaths [2]. This new entity has almost the same features as the pediatric vascular inflammatory syndrome Kawasaki disease (KD). One of those studies found a high proportion of children of African ancestry among the Kawasaki-like disease [3]. We report here the first five cases of Kawasaki-like disease observed for two months, since October 2020, in a university hospital in Casablanca, Morocco. Our study aims to highlight the monitoring requirement in children living in a family cluster and to identify the first symptoms of multisystem inflammatory syndromes.

## Case presentation

Patient 1

A three-year-old boy presented to our emergency department with a seven-day history of high fever, mild cough, complicated three days prior to his admission by asthenia, and generalized skin rash. His previous medical history was unremarkable except for a SARS-CoV-2 infection in his parents and grand-parents three weeks ago. On admission, physical examination revealed fever of 40°C, irritability, right cervical adenitis measuring 2 cm, conjunctivitis, erythema, and cracked lips, skin rash, erythema, and edema of the hands and feet, petechial elements, and prolonged capillary refill time. Otherwise, the respiratory rate and heart rate were normal. Blood tests revealed significant lymphocytopenia, mild thrombocytopenia, and elevated levels of inflammatory markers (Table 1). A nasopharyngeal swab was negative for SARS-CoV-2, while the serology was positive for immunoglobulin G (IgG). A chest CT scan, abdominal ultrasound exam, and echocardiography were normal. The patient was diagnosed with a complete KD and COVID-19 infection. Intravenous immunoglobulin (IVIG), a high dose of aspirin, and methylprednisolone at 2 mg/kg/day were applied. The outcome was favorable with defervescence as the second day, prompt general improvement, and normalization of blood tests. A follow-up echocardiogram performed 15 days later was normal.

**Table 1:** Laboratory finding of all patients at admission

	Normal values	Case 1	Case 2	Case 3	Case 4	Case 5
Erythrocyte count (10¹²/L	3.7 - 5.5	4.03	3.47	4.58	4.49	4.54
Hemoglobin (g/dL)	10.9 - 13.7	10.2	10.2	12.2	12.9	11.7
Hématochrit (%)	30 - 41	30.7	27.7	35.5	36	34.1
Mean cell volume (MCV, fL)	68 - 86	76.2	79.8	77.5	80.2	75.1
Mean cell hemoglobin (MCH, pg)	23 - 31	25.3	29.4	26.6	28.7	25.8
Mean corpuscular hemoglobin concentration (MCHC, g/Dl)	30.0 - 37.4	33.2	36.8	34.4	35.8	34.3
Platelet count (10ᶟ/mm)	200 - 550	130	27000	107	303	8
Blood leukocyte (count/mm)	6000 - 17500	8710	9770	6170	7750	6880
Lymphocyte (%)	-	22.8	8	5.3	21.5	24
Lymphocyte count (10ᶟ/mm)	1.5 - 7	1.99	0.78	0.33	1.67	1.65
Neutrophil (%)	-	69	87.3	92.8	62	65.2
Neutrophil count (10ᶟ/mm)	1.0 - 8.5	6.01	8530	5.73	4.81	4.49
Monocyte (%)	-	5.6	2.3	1.5	13,9	10.3
Monocyte count (10ᶟ/mm)	0.2 - 1.0	0.49	0.22	0.09	1.08	0.71
Basophils (%)	-	0.2	0.3	0.2	0.3	0.1
Basophil count (10ᶟ/mm)	< 0.1	0.02	0.03	0.01	0.02	0.01
C-reactive protein level (mg/L)	< 8	145.81	344.57	400.18	97.47	251
Procalcitonine (ng/ml)	< 0.5	>100	34.68	20.8	0.201	24
Creatinine (mg/L)	7 - 13	3.8	18.2	5.6	3.1	4.74
Blood urea nitrogen (g/L)	0.15 - 0.45	0.36	1.22	0.25	0.23	0.24
Aspartate aminotransferase (U/L)	5 - 34	109.5	25	27.8	18.2	20
Alanine aminotransferase (U/L)	< 55	135	34	15.6	5.6	10
Lactic dehydrogenase (UI/L)	130 - 340	632	276	439	164	257
Fibrinogen (g/L)	-	-	5.47	-	-	-
D-dimer (ng/ml)	< 500	3470	3397.71	6007	480	1962.59
Prothrombin time (%)	70 - 100	-	98	75	-	88
Activated partial thromboplastin time (sec)	-	-	29.4	47.6	-	28.2
International normalized ratio (INR)	-	-	1.01	1.22	-	1.08
Serum ferritin (ng/mL)	15 - 80	1084	1667	2597	355	373.21
Creatine phosphokinase (UI/L)	< 170	-	-	95	-	26
Sodium (mEq/L)	136 - 145	132	127	130	136	135
Potassium (mEq/L)	3.5 - 5.1	3.5	5.0	3.1	4.2	3.9
Serum chloride (mEq/L)	98 - 107	97	90	89	94	106
Troponin I (ng/mL)	< 0.03	-	0.038	0.002	-	-
Sedimentation rate (mm)	< 13	25	125	84	75	90

Patient 2

A seven-year-old boy arrived in our emergency department with a five-day history of fever, complicated the day of his admission by acute abdominal pain, tenderness, nausea, vomiting, and low urine output. His previous medical history was unremarkable. On admission, a physical examination revealed fever of 38,5°C, irritability, disorientation, bilateral conjunctivitis, cheilitis, edema of the hands, and feet, tachycardia, heart rate was 140 beats/min, low blood pressure at 72/38 mmHg, while the respiratory rate was normal. Besides, an increased abdominal wall rigidity, distension, and generalized ileus were noted.

**Figure 1:**
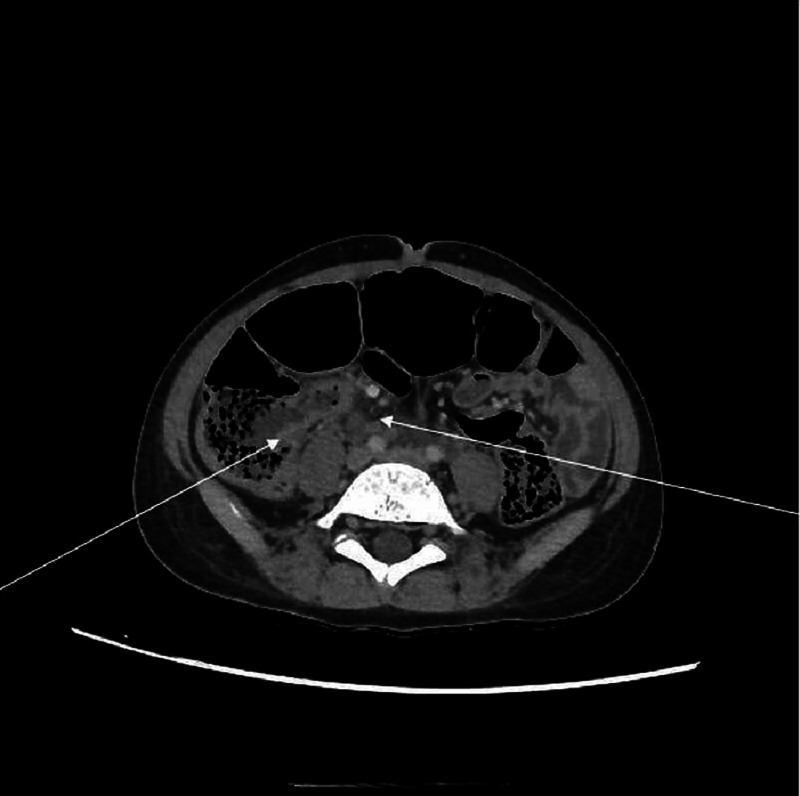
Abdominal CT scan showing Ileitis and abdominal deep adenopathies Right arrow: Ileitis Left arrow: abdominal deep adenopathies

Blood tests revealed anemia, lymphocytopenia, deep thrombocytopenia, hypertriglyceridemia, significantly increased ferritinemia levels and other inflammatory markers, and increased serum creatinine levels (Table 1). A chest CT scan revealed a double focus of bilateral pneumonia. An abdominal CT scan revealed splenomegaly, ileitis, and multiple deep abdominal adenopathies but without peritoneal fluid (Figure 1). The blood cultures and the urinalysis were steril. A nasopharyngeal swab specimen was negative, while serology was positive for SARS-CoV-2. The echocardiography found dilation of a left coronary artery at 5 mm and dilation of the right coronary artery at 4 mm with minor aortic valvular regurgitation and minimal pericardial effusion. The patient was diagnosed with multisystem inflammatory syndrome associated with SARS-CoV-2 infection. Intravenous immunoglobulin (IVIG), a high dose of aspirin, and methylprednisolone at 2 mg/kg/day were applied besides a supportive treatment. The outcome was favorable with defervescence as the third day, prompt general improvement, and normalization of blood tests as the fourth day. A follow-up echocardiogram performed 15 days later showed the same finding.

Patient 3

**Figure 2:**
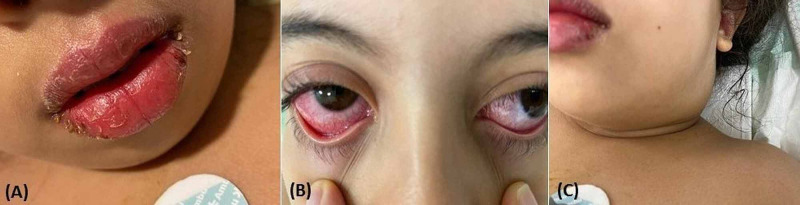
Kawasaki-like disease (A) Cheilitis (B) Bilateral conjunctivitis (C) Cervical lymph adenopathy

A nine-year-old girl arrived in our emergency department with a five-day history of fever and diarrhea. Her previous medical history was unremarkable. A physical examination revealed a fever of 40°C, pharyngitis, irritability, bilateral cervical swelling, bilateral conjunctivitis, erythema, cracked lips, and generalized skin rash (Figure 2). Heart rate was 151 beats/min, and the respiratory rate was normal. The sensitivity of the left hypochondrium but without hepatosplenomegaly.

Blood tests revealed severe lymphocytopenia, thrombocytopenia, and significantly increased levels of ferritin and other inflammatory markers (Table 1). A chest CT scan and abdominal ultrasonography were normal. The echocardiogram revealed a dilatation of the common coronary trunk. The cervical ultrasonography revealed bilateral lymphadenitis. The blood cultures did not identify any bacteria. A nasopharyngeal swab specimen was negative for SARS-CoV-2, while the serology was positive. The patient was diagnosed with complete KD triggered by a COVID-19 infection. Intravenous immunoglobulin (IVIG), a high dose of aspirin, and methylprednisolone at 2 mg/kg/day were applied. The outcome was favorable with defervescence on the third day, prompt general improvement, and normalization of blood tests on the fifth day. A follow-up echocardiogram performed 15 days later found the same image of the dilatation of the common coronary trunk.

Patient 4

**Figure 3:**
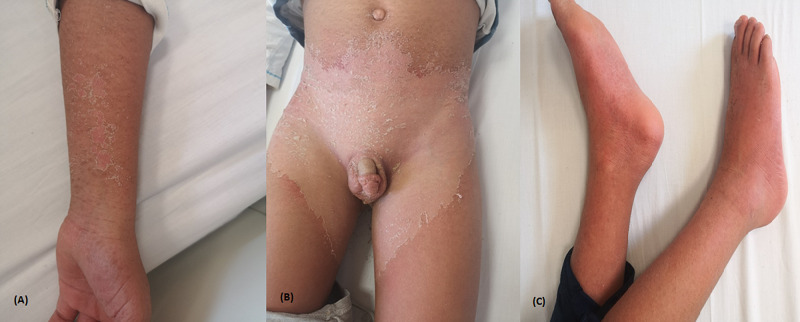
Kawasaki-like disease (A) Limb exanthema with desquamation (B) Perineal exanthema with desquamation (C) Erythema with edema of the palms and foot soles

A ten-year-old boy presented to our emergency department with an eight-day history of high fever, anorexia, generalized skin rash, and impairment of his general condition. His previous medical history was unremarkable except for SARS-CoV-2 infection in his parents five days ago. On admission, physical examination revealed fever of 40°C, asthenia, obstructive rhinitis, bilateral conjunctivitis, stomatitis, erythema, and cracked lips, skin rash, erythema, and edema of the hands and feet. An erythema-squamous rash on the chest, limbs, and perineum (Figure 3). Besides, he presented bilateral cervical adenitis. Otherwise, the respiratory rate and heart rate were normal. Blood tests revealed mild inflammatory syndrome, lymphocytopenia, mild thrombocytopenia, and elevated levels of inflammatory markers (Table 1). A nasopharyngeal swab was negative for SARS-CoV-2, the serology was negative too. A chest CT scan and echocardiography were normal. The patient was diagnosed with a complete KD and COVID-19 infection. Intravenous immunoglobulin (IVIG), a high dose of aspirin, and methylprednisolone at 2 mg/kg were applied. The outcome was favorable with defervescence as the second day, prompt general improvement, and normalization of blood tests. A follow-up echocardiogram performed 15 days later was normal.

Patient 5

**Figure 4:**
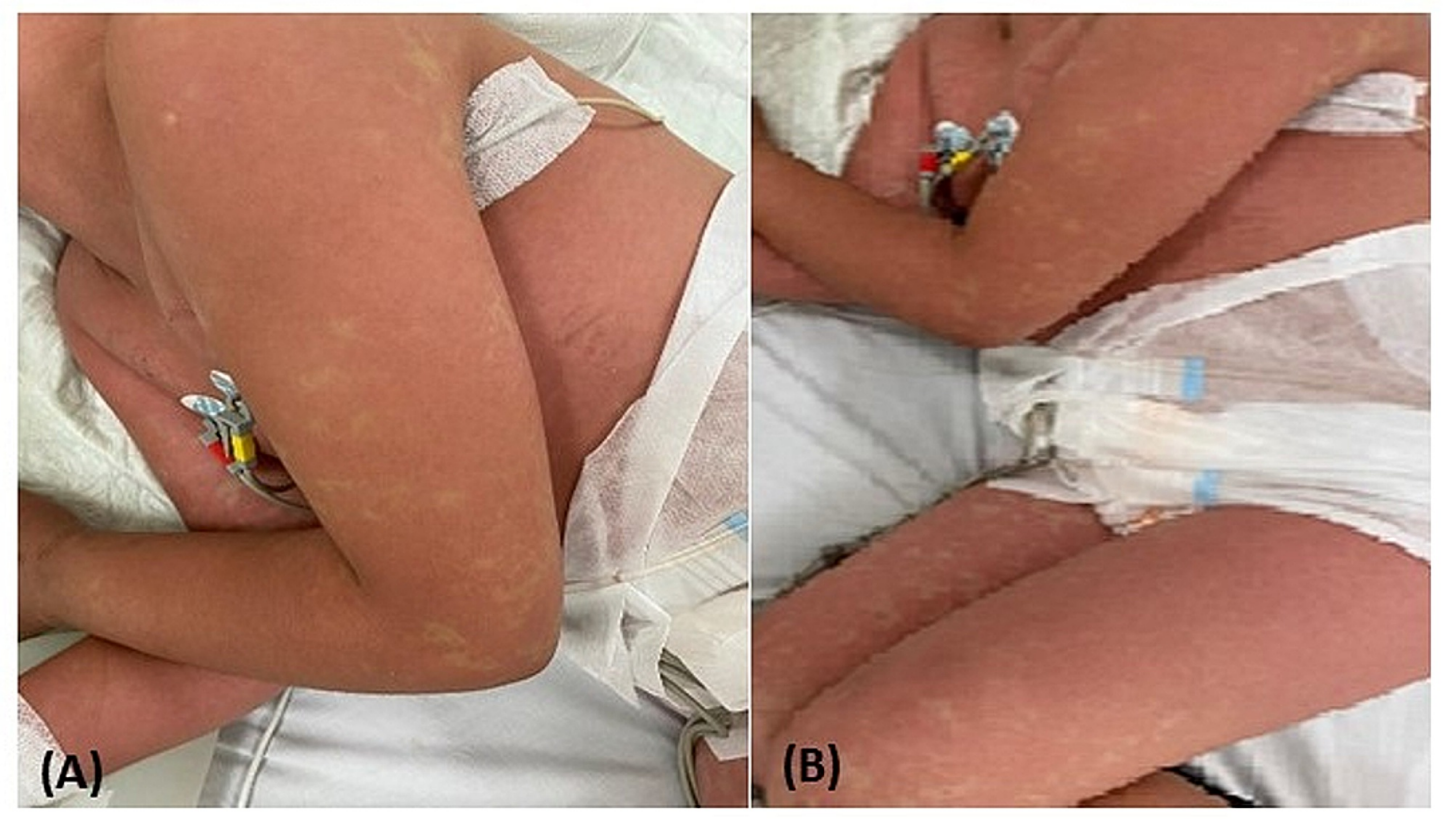
Kawasaki-like disease (A), (B): Generalized skin rash

A 10-years-old girl presented to our emergency department with a five-day history of high fever, asthenia, complicated on the day of her admission by a generalized skin rash. She had a history of ischemic stroke due to protein S transient insufficiency five years ago. On admission, physical examination revealed a fever of 40°C, asthenia, bilateral conjunctivitis, eyelid erythema, and edema (Figure 4). Stomatitis, erythema, and cracked lips, skin rash, erythema, and edema of the hands and feet. Besides, she presented bilateral cervical adenitis. Otherwise, the respiratory rate and heart rate were normal. Blood tests revealed mild inflammatory syndrome lymphocytopenia, severe thrombocytopenia, and elevated levels of inflammatory markers (Table 1). A nasopharyngeal swab was positive for SARS-CoV-2, the serology was positive too. A chest CT scan and echocardiography were normal. The patient was diagnosed with a complete KD and COVID-19 infection. Intravenous immunoglobulin (IVIG), a high dose of aspirin, and methylprednisolone at 2 mg/kg were applied. The outcome was favorable with defervescence as the second day, prompt general improvement, and normalization of blood tests. A follow-up echocardiogram performed 15 days later was normal.

## Discussion

In the midst of the COVID-19 pandemic, we are seeing an impressive increase in generalized inflammatory morbidities affecting patients of all ages around the world. An epidemiological study conducted in China showed that 90% of 731 laboratory-confirmed COVID-19 patients under the age of 18 had an asymptomatic, mild, or moderate infection, making it relatively mild compared to adult infection [4].

KD is an acute autoimmune vasculitis characterized by extensive inflammation of small and medium-sized vessels. It almost exclusively affects children [5]. The diagnosis of “classic” KD is based on clinical criteria; it’s considered in patients presenting with fever for more than five days together with at least four out of five clinical criteria in the absence of an alternate diagnosis. One of the most severe complications of Kawasaki disease is coronary artery aneurysm [6]. Without adequate treatment, 20% of untreated children can develop a coronary artery aneurysm, posing a significant risk of thrombosis and myocardial infarction later in life. Large aneurysms can rupture and lead to death. Severe cases of KD may present with a state of shock (Kawasaki shock disease syndrome) similar to what has been described currently in association with COVID-19 [7].

The cause of KD remains unknown, although half a century has passed since KD's first case was reported. The most accepted hypothesis supports an aberrant response of the immune system to one or more unidentified pathogens in genetically predisposed patients. Some evidence suggests an infectious trigger, particularly viral, with the winter-spring seasonality of the disease [8]. Several studies have shown a causal link between KD and certain viruses such as rhinovirus, parainfluenza virus, respiratory syncytial virus, adenovirus [9], human coronavirus 229E (HCoV-229E) [10], and the New Haven coronavirus (HCoV-NH) [11]. This suggests that the coronavirus family, including SARS-CoV-2 being a particularly virulent strain, could elicit a potent immune response in the host and therefore represent one of the triggers for Kawasaki disease. Indeed, an exaggerated systemic inflammatory response (cytokine storm) leading to vascular endothelial damage and immune-mediated tissue damage is described in both conditions [12].

An alert issued on May 4, 2020, by the New York Department of Health, reported the occurrence of 15 cases of patients aged from two to 15 years with typical symptoms of Kawasaki disease and inflammatory shock Kawasaki disease (Kawasaki-like) [13]. Similar cases in pediatric patients have also been found in Italy at the Bergamo hospital, where more than 20 children have been admitted with hyperinflammatory shock similar to that of Kawasaki syndrome. An observational study from the Bergamo province in Italy, which had a high rate of SARS-CoV-2 infections at that time, reported a 30-fold increased monthly incidence of KD in a cohort of children from February 18, 2020, to April 20, 2020, compared with a cohort of patients from the previous five years [14]. The same hyperinflammatory shock in children has also been observed in France, Spain, Portugal, and Pakistan [10, 15]. Interestingly, some series noted frequent gastrointestinal symptoms, including bloodless diarrhea, abdominal pain, severe vomiting, and imaging revealed ascites and ileitis [7, 14]. These results show that COVID19 is not always benign in children. Increasingly serious post-infectious forms are described in recent literature. Rigorous monitoring for several weeks is required in any positive child or living in a family cluster.

Our series had a median age of 7.8 years, which is not usual in the classic KD, which often affects children under three years. Our result does not differ from an Italian and French KD series with COVID-19, where the median age was 7.5 years and 7.9 years, respectively [14, 3]. From a clinical perspective, four cases presented complete and moderate criteria for Kawasaki disease, while the fifth case (patient 2) had a hyperinflammatory shock with organ failure and hemodynamic instability. Two cases (patients 1 and 2) presented with distension and diffuse abdominal pain accompanied by watery diarrhea only in the first case. All cases received intravenous immunoglobulins and high doses of aspirin with a rapidly favorable outcome in four cases. One of the cases (patient 3) presented with a very disturbed inflammatory assessment and resistance to the first course of intravenous immunoglobulins and a need for additional corticosteroids and a second treatment with immunoglobulins. The same patient and the second patient had coronary dilation on echocardiography.

Evidence of contact with the virus was confirmed by the presence of antibodies against SARS-CoV-2 in four of five patients. Two of the four positive cases had elevated IgG anti-COVID-19 antibodies. The positivity of IgG antibodies suggests a late onset of the disease compared to the primary infection, suggesting that the development of Kawasaki disease is more likely to be the result of a post-viral immunological reaction. The negative patient was tested just after an infusion of immunoglobulins, which can neutralize antibodies against SARS-CoV-2. However, the diagnosis of SARS-CoV-2 infection can't be ruled out in him because both parents were positive of SARS-CoV-2. Our study included only five cases over a two-month period, limiting its reliability due to the small sample size. Further large-scale studies with genetic studies are needed to prove this association between pediatric COVID-19 infection and KD.

## Conclusions

These clinical cases support the already published observations of the post-COVID-19 inflammatory syndrome in children in the Moroccan context and confirm that this syndrome can be life-threatening. In conclusion, if COVID-19 infection is manifested in adults with respiratory symptoms of different degrees of severity, in children, it may present as a post-COVID-19 inflammatory syndrome with similarities to Kawasaki disease and macrophage activation syndrome with poor prognosis. These findings are intended to change the paradigm regarding the screening and follow-up of contact cases among children and even to consider a strategy for childhood vaccination.
